# Smoking and obesity are associated with chronic hand eczema and severity of hand eczema: Data from the Dutch general population

**DOI:** 10.1111/cod.14110

**Published:** 2022-04-05

**Authors:** L. Loman, K. Politiek, M. L. A. Schuttelaar

**Affiliations:** ^1^ Department of Dermatology University of Groningen University Medical Center Groningen Groningen the Netherlands; ^2^ Medical Center Leeuwarden Department of Dermatology Leeuwarden the Netherlands

**Keywords:** general population, hand dermatitis, hand eczema, lifestyle factors, obesity, smoking, stress

## INTRODUCTION

With an increasing focus on preventive and personalized treatment programs in medicine, lifestyle behavior also becomes an important research topic in skin diseases. Several studies reported on the association between lifestyle factors and hand eczema (HE) before, and recently the association between HE and lifestyle factors was also investigated in a large sample of the Dutch general population.^1^ However, only a small subset of the previous studies included severity of HE as outcome measure,^2^, ^3^, ^4^ and only a few of them focused specifically on chronic hand eczema (CHE). Therefore, the aim of the current study was to assess the association between lifestyle factors and HE from the same large sample of the Dutch general population, however, this time with a focus on prevalence of CHE and severity of HE.

## METHODS

This cross‐sectional questionnaire‐based study used data from the Lifelines Cohort Study, a multi‐disciplinary prospective population‐based cohort study examining the health and health‐related behaviors of 169 729 persons living in the North of The Netherlands.^5^ At baseline, 2006‐2013, information on lifestyle factors was collected and an additional questionnaire including questions regarding HE was sent out to all adults (*n* = 135 950) in 2020. In total, 58 198 participants responded (42.8%) of which 57 046 were 18 years or older at baseline and were included in the present analysis. Institutional review board approval was obtained at the University Medical Center Groningen, and all participants provided informed consent. Details on definitions and categorization of CHE and all lifestyle factors has been published before and can be found in Appendix S1; Table S1.^1^, ^6^ Binary logistic regression models were performed with CHE in the past year vs never HE, and severe‐to‐very severe HE vs almost clear‐to‐moderate HE at worst in the past year, as the dependent variables. Multivariate analysis were adjusted for age, sex, atopic dermatitis (AD), and wet activities. *p*‐Values of <0.05 were considered to be statistically significant.

## RESULTS

Data regarding prevalence and severity of HE for the whole study population have been described previously.^6^ The 1‐year‐prevalence of CHE in the current study population was 4.6%. In total, 7.4% of all subjects with HE in the past year reported having severe or very severe HE at worst in the past year, resulting in a 1‐year prevalence of severe‐to‐very severe HE at worst in the past year of 0.5%. Multivariate analysis showed a positive association between being female,AD, exposure to wet activities, and CHE. In addition, smoking, especially smoking ≥8 cigarettes/day, a smoking history of ≥15 pack‐years, stress, overweight and obesity, and a higher waist circumference (all at baseline) were also positively associated with having CHE in the past year. Age showed a negative association with CHE in the past year. Furthermore, the highest category of physical activity at baseline showed a negative association with CHE in the past year (Table 1).

**TABLE 1:** Patient characteristics and lifestyle factors for the total study population, stratified by subjects with chronic hand eczema, subjects without hand eczema ever, and severity of hand eczema

	Total *n* = 57.046 *n* (%)	CHE *n* = 2.649, *n* (%)	HE never *n* = 48.496 *n* (%)	Severe‐very severe HE *n* = 309, *n* (%)	Almost clear‐moderate HE *n* = 3845, *n* (%)	CHE vs HE never		Severe‐very severe HE vs almost clear‐moderate HE	
						Adjusted OR (95% CI)^a^	*p*‐value	Adjusted OR (95% CI)^a^	*p*‐value
**Sex (female)**	34.396 (60.3)	1.872 (70.7)	28.273 (58.3)	229 (74.1)	2699 (70.2)	**1.31 (1.19–1.44)**	**<0.001**	0.91 (0.68–1.23)	0.55
**Age (years)**	55.8 ± 12.2	51.3 ± 11.5	56.2 ± 12.2	49.8 ± 12.2	51.4 ± 11.5	**0.98 (0.97**–**0.98)**	**<0.001**	**0.98 (0.97**–**0.99)**	**<0.001**
**Atopic dermatitis**	5.145 (9.2)	977 (38.8)	2.730 (5.7)	179 (62.4)	1154 (31.4)	**9.11 (8.31**–**10.00)**	**<0.001**	**3.66 (2.83**–**4.74)**	**<0.001**
**Wet activities**	13.299 (24.6)	854 (33.9)	10.689 (23.2)	124 (42.5)	1185 (32.3)	**1.37 (1.24**–**1.50)**	**<0.001**	**1.42 (1.09**–**1.85)**	**<0.001**
Smoking									
Never	26.343 (47.0)	1.256 (48.2)	22.376 (46.9)	126 (41.9)	1879 (50.0)	1	–	1	–
Former	20.521 (36.6)	827 (31.7)	17.608 (36.9)	84 (27.9)	1213 (32.3)	1.07 (0.96–1.18)	0.21	1.20 (0.87–1.66)	0.27
Current	9.454 (16.8)	524 (20.1)	7.905 (16.5)	93 (30.7)	689 (18.2)	**1.14 (1.02**–**1.28)**	**0.03**	**2.10 (1.54**–**2.86)**	**<0.001**
0.1–7 cig/day	3.640 (6.5)	184 (7.1)	3.085 (6.5)	28 (9.3)	248 (6.6)	1.00 (0.84–1.19)	0.99	**1.74 (1.10**–**2.77)**	**0.02**
≥8 cig/day	5.557 (9.9)	328 (12.6)	4.602 (9.7)	63 (20.9)	417 (11.1)	**1.25 (1.08**–**1.43)**	**0.002**	**2.33 (1.63**–**3.33)**	**<0.001**
<15 packyears	20.047 (36.7)	901 (35.6)	16.970 (36.5)	113 (38.8)	1311 (35.5)	1.04 (0.95–1.15)	0.38	**1.44 (1.07**–**1.92)**	**0.02**
≥15 pack‐years	8.271 (15.1)	377 (14.9)	7.099 (15.3)	52 (17.9)	498 (13.5)	**1.26 (1.10**–**1.45)**	**0.001**	**1.99 (1.34**–**2.94)**	**0.001**
Stress									
LTE									
0	25.042 (44.7)	1.076 (41.3)	21.520 (45.2)	114 (37.9)	1578 (41.8)	1	–	1	–
1	15.665 (28.0)	741 (28.5)	13.238 (27.8)	88 (29.2)	1087 (28.8)	**1.08 (0.97**–**1.20)**	**0.17**	1.05 (0.77–1.43)	0.78
2	8.905 (15.9)	464 (17.8)	7.489 (15.7)	53 (17.6)	650 (17.2)	**1.18 (1.04**–**1.34)**	**0.009**	1.07 (0.74–1.56)	0.71
≥3	6.424 (11.5)	322 (12.4)	5.373 (11.3)	46 (15.3)	463 (12.3)	1.14 (0.99–1.31)	0.07	1.34 (0.91–1.96)	0.14
LDI									
0	12.708 (22.7)	377 (14.5)	11.342 (23.8)	34 (11.3)	569 (15.1)	1	–	1	–
1–2	21.989 (39.3)	966 (37.1)	18.869 (39.6)	102 (33.9)	1400 (37.1)	**1.19 (1.05**–**1.36)**	**0.009**	1.08 (0.70–1.67)	0.72
3–4	12.498 (22.3)	701 (26.9)	10.328 (21.7)	97 (32.2)	987 (26.1)	**1.37 (1.19**–**1.58)**	**<0.001**	1.34 (0.86–2.08)	0.19
≥5	8.825 (15.8)	558 (21.4)	7.069 (14.8)	68 (22.6)	820 (21.7)	**1.35 (1.16**–**1.56)**	**<0.001**	1.12 (0.70–1.78)	0.63
BMI (kg/m^2^)									
<25	26.740 (46.9)	1.239 (46.8)	22.625 (46.7)	142 (46.0)	1867 (48.6)	1	–	1	–
25–30	22.155 (38.9)	980 (37.0)	19.042 (39.3)	102 (33.0)	1394 (36.3)	**1.11 (1.01**–**1.22)**	**0.04**	1.00 (0.75–1.34)	0.98
>30	8.132 (14.3)	430 (16.2)	6.813 (14.1)	65 (21.0)	584 (15.2)	**1.23 (1.08**–**1.39)**	**0.01**	**1.57 (1.12**–**2.20)**	**0.01**
Waist circumference (cm)									
≤80	13.188 (23.1)	711 (26.8)	10.919 (22.5)	92 (29.8)	1037 (27.0)	1	–	1	–
>80–90	17.593 (30.9)	783 (29.6)	14.911 (30.8)	81 (26.2)	1198 (31.2)	1.04 (0.93–1.17)	0.49	0.87 (0.62–1.22)	0.41
>90–100	15.765 (27.6)	691 (26.1)	13.594 (28.0)	71 (23.0)	982 (25.5)	**1.23 (1.09**–**1.40)**	**0.001**	0.93 (0.64–1.36)	0.72
>100–110	7.439 (13.0)	323 (12.2)	6.452 (13.3)	46 (14.9)	430 (11.2)	**1.23 (1.05**–**1.44)**	**0.01**	1.46 (0.95–2.24)	0.08
>110	3.042 (5.3)	141 (5.3)	2.605 (5.4)	19 (6.1)	198 (5.1)	**1.31 (1.06**–**1.62)**	**0.01**	1.40 (0.80–2.45)	0.25
Physical activity (min/wk)									
No MVPA (0)	3.363 (6.4)	191 (7.8)	2.831 (6.4)	28 (10.2)	240 (6.7)	1	–	1	–
MVPA–T1 (>0–240)	16.403 (31.3)	844 (34.6)	13.710 (30.8)	82 (29.9)	1288 (36.0)	0.88 (0.74–1.05)	0.16	**0.45 (0.28**–**0.72)**	**0.001**
MVPA–T2 (>240–725)	16.348 (31.2)	740 (30.3)	13.833 (31.0)	73 (26.6)	1123 (31.4)	0.84 (0.70–1.00)	0.05	**0.47 (0.29**–**0.77)**	**0.002**
MVPA–T3 (>725)	16.356 (31.2)	667 (27.3)	14.199 (31.9)	91 (33.2)	931 (26.0)	0.97 (0.80–1.17)	0.74	0.78 (0.48–1.27)	0.32
No VPA (0)	8.127 (15.5)	420 (17.2)	6.884 (15.4)	58 (21.2)	551 (15.4)	1	–	1	–
VPA–T1 (>0–120)	15.857 (30.2)	791 (32.4)	13.329 (29.9)	95 (34.7)	1171 (32.7)	0.90 (0.79–1.03)	0.12	**0.65 (0.45**–**0.94)**	**0.02**
VPA–T2 (>120–290)	13.715 (26.1)	636 (26.0)	11.657 (26.2)	55 (20.1)	968 (27.0)	0.89 (0.78–1.02)	0.10	**0.51 (0.34**–**0.77)**	**0.001**
VPA–T3 (>290)	14.771 (28.2)	595 (24.4)	12.703 (28.5)	66 (24.1)	892 (24.9)	**0.83 (0.72**–**0.95)**	**0.008**	**0.63 (0.42**–**0.93)**	**0.02**

*Note*: Adjusted odds ratios (ORs) with 95% confidence intervals (CIs) are presented. *p*‐values <0.05 are shown in bold. Data on HE, CHE, severity of HE, age, AD, and exposure to wet activities were part of the add‐on questionnaire; all other variables were included in the baseline assessment. Exact definitions for each variable were published previously and can be found in Appendix S1; Table S1.^1^, ^6^

Abbreviations: BMI, body mass index; CHE, chronic hand eczema; CI, confidence interval; cig, cigarettes; cm, centimeter; HE, hand eczema; kg/m^2^, kilogram per square meter; LDI, Long‐term Difficulties Inventory; LTE, List of Threatening Experiences; min/wk, minutes/week, MVPA, moderate and vigorous physical activity; n, number; OR, odds ratio; T, tertile; VPA, vigorous physical activity.

^a^ Adjusted for: age, sex, atopic dermatitis, and exposure to wet activities.

For severe‐to‐very severe HE in the past year, multivariate analyses showed a negative association between age and severity of HE, and a positive association between AD, exposure to wet activities, smoking (regardless amount of cigarettes or pack‐years), obesity (both at baseline), and severity of HE. In addition, a negative association between almost all categories of physical activity was found (Table 1).

## DISCUSSION

Regarding the lifestyle factors, smoking and obesity were associated with both the self‐reported 1‐year prevalence of CHE and severity of HE, which is in line with the previously published results of the association between lifestyle factors and having HE in the past year in the same study population.^1^ Reporting less physical activity was associated particularly with severe‐to‐very severe HE. Stress and being overweighed were only positively associated with CHE.

Some previous studies reporting results on the association between lifestyle factors and HE, also focused on severity of HE.^2^, ^3^, ^4^ For example, a large prospective cohort study in 1608 patients with occupational HE demonstrated during clinical follow‐ups over 3 years that tobacco smoking was associated with severity of HE at all time points.^4^ However, a cross‐sectional clinical study, including 109 subjects with physician‐diagnosed HE, reported no association between smoking, stress, body mass index (BMI), physical activity, alcohol consumption, and severity of HE after adjustment for possible confounders.^2^ Severity of HE in both studies was assessed by the Osnabrück Hand Eczema Severity Index (OHSI). Another cross‐sectional occupational study of 773 subjects with self‐reported HE, found a positive association between smoking and severity of HE and no association between stress or BMI and severity of HE. The self‐administered photographic guide was used to assess current severity.^3^ These results are partly in line with the current study and conflicts might be explained by the varying study setting and methods of severity assessment and diagnosis between the studies. In addition, the current study design needs to be taken into consideration, were data from lifestyle factors was collected several years before the questionnaire regarding CHE and severity of HE was send out, which could have altered the results due to fluctuations of some lifestyle factors.

In conclusion, smoking and obesity were associated with CHE and severity of HE. Replication of these results in an independent cohort will be important to support these findings. Ideally, future research should include the evaluation of the effect of lifestyle interventions in daily practice. However, the effect of lifestyle interventions on CHE and severity of HE might be influenced by the persistent effect of the lifestyle factor, even after cessation of smoking or weight reduction. Therefore, further research will be needed to evaluate if secondary‐prevention strategies in clinical practice are of added value when counseling patients with HE.

## AUTHOR CONTRIBUTIONS

All authors have participated sufficiently to take public responsibility for appropriate portions of the work. **L. Loman:** Conceptualization (equal); Data curation (lead); Formal analysis (lead); Investigation (equal); Methodology (equal); Project administration (lead); Visualization (equal); Writing original draft (lead); Writing‐review, and editing (supporting). **K. Politiek:** Supervision (supporting); Writing‐review, and editing (equal). **M. L. A. Schuttelaar:** Conceptualization (equal); Funding acquisition (lead); Investigation (equal); Methodology (equal); Resources (lead); Supervision (lead); Visualization (equal); Writing‐review, and editing (equal).

## CONFLICTS OF INTEREST

M.L.A. Schuttelaar received consultancy fees from Sanofi‐Genzyme and Regeneron Pharmaceuticals; and is an advisory board member for Sanofi‐Genzyme, Regeneron Pharmaceuticals, Pfizer, LEO Pharma, and Lilly. No other conflicts are reported.

## Supporting information


**Appendix S1** Supporting information.
