# Supplementary material for: Smoking and obesity are associated with chronic hand eczema and severity of hand eczema: Data from the Dutch general population
Source: Contact Dermatitis. 2022 Apr 5;87(1):103–6. doi: 10.1111/cod.14110 (PMC9324152; doi:10.1111/cod.14110)
Supplement: Supplementary file 1 — Appendix S1 Supporting information. [file COD-87-103-s001.docx]

**Smoking and obesity are associated with chronic hand eczema and severity of hand eczema: data from the Dutch general population**

L. Loman^1^, K. Politiek^2^, M.L.A. Schuttelaar^1^

Names of the institutions:

1. University of Groningen, University Medical Center Groningen, Department of Dermatology, Groningen, the Netherlands
2. Medical Center Leeuwarden, Department of Dermatology, Leeuwarden, the Netherlands

**Supplementary material**

**Part of this supplemental material was previously published by Loman et al. as online supplement.**^1^

**Table S1.** Questions used in the current study, including used definitions to categorize variables, and relevant references.

| **Topic** | **Question and response options**  **English translation** | **Categorization** | **References** |
| --- | --- | --- | --- |
| **Lifetime prevalence of hand eczema** | Hand eczema is a skin disease of the hands. Symptoms are redness, desquamation/dry skin and sometimes vesicles and/or fissures. Hand eczema can cause itch and/or pain.  Have you ever (now or in the past) had hand eczema?   - Yes - No | - Yes (1) - No (0) | Nordic Occupational Skin Questionnaire (NOSQ)-2002; question D1, adjusted.^2^ |
| **1-year prevalence of hand eczema** | Have you had hand eczema in the past 12 months?   - Yes - No | - Yes (1) - No (0) | NOSQ-2002; question D1, adjusted.^2^ |
| **Age** | What is your year of birth? |  | None. |
| **Sex** | What is your sex?   - Male - Female | - Male (1) - Female (2) | None. |
| **Atopic dermatitis** | Have you ever been diagnosed with atopic dermatitis or atopic eczema by a doctor?   - Yes - No | - Yes (2) - No (1) | Barbarot et al. 2018; question P1.^3^ |
| **Exposure to wet activities** | Three questions about exposure to wet activities were included, they were separately asked for activities at work and at home, resulting in six variables regarding exposure to wet activities.  1. On an average day, how many hours do your hands come into direct contact with water, fluids and/or moist products? (for example during washing your hands)   - Never (0) - Less than ½ hour (1) - ½-1 hour (2) - 1-2 hours (3) - More than 2 hours (4)   2. On an average day, how many hours do you wear gloves that are impermeable to fluids?   - Never (0) - Less than ½ hour (1) - ½-1 hour (2) - 1-2 hours (3) - More than 2 hours (4)   3. On an average day, how often do you wash your hands?   - Never (0) - Less than 5 times (1) - 5-10 times (2) - 10-20 times (3) - More than 20 times (4) | Exposure to wet activities was defined as minimally two or more from answer category 3, at least one from answer category 4. | Questions based on the German Technische Regeln für Gefahrstoffe (TRGS) 401 criteria and an article by Behroozy et al.^4,5^ |
| **Smoking** | Several questions regarding smoking habits were included:  1. Have you ever smoked for as long as a year?   - Yes - No   2. Do you smoke now or have you been smoking in the last month?  3. Did you quit smoking?   - Yes - No   4. How many cigarettes/cigarillos/cigars/pipe tobacco do you smoke now on average per day? | - Never-smokers (question 1 = no) - Former smokers (question 3 = yes and answer question 4 = 0) - Current smokers (question 2 = yes and answer question 4 > 0)   Current smokers were further categorized based on their answer on question 4:  < 8 cigarettes/cigarillos/cigars/pipe tobacco per day  ≥8 cigarettes/cigarillos/cigars/pipe tobacco per day | None. |
| **Pack years** | How much did you smoke up till now?  From age .. till age .. I smoked … cigarettes/cigarillos/cigars/pipe tobacco per day | One pack year was defined as 20 cigarettes per day for 1 year, cigars were regarded as 3 cigarettes.  Packyears were further categorized:   - < 15 pack years - ≥ 15 pack years | None. |
| **Stress - List of Threatening Experiences (LTE)** | In the next questionnaire 12 unpleasant events are listed. Please indicate if you have experienced these events in the past 12 months.   - Yes - No   1. You yourself suffered a serious illness, injury or an assault  2. A serious illness, injury or assault happened to a close relative  3. Your parent, child or spouse died  4. A close family friend or another relative (aunt, cousin, grandparent) died  5. You had a separation due to marital difficulties  6. You broke off a steady relationship  7. You had a serious problem with a close friend, neighbor or relative  8. You became unemployed or you were seeking work unsuccessfully for more than 1 month  9. You were sacked from your job  10. You had a major financial crisis  11. You had problems with the police and a court appearance  12. Something you valued was lost or stolen | - 0 events (1) - 1 event (2) - 2 events (3) - ≥ 3 events (4) | LTE^6,7^ |
| **Stress - Long-term Difficulties Inventory (LDI)** | Below is a list of various aspects of life. We would like to know how you experience these aspects with respect to  difficulty and stress in the past 12 months and the successive age categories. Fill the circle in on every row which corresponds to how you felt:   - Not stressful (0 points) - Slightly stressful (1 point) - Very stressful (2 points)   1. Housing (e.g. house is too small, could not find a house, noise problems)  2. Work (e.g. too exacting, conflicts with boss, (threatening) resigned or sacked)  3. Relationship with friends or good acquaintances (e.g. arguments, not enough support)  4. Relationship with partner (e.g. jealousy, conflicts, doubts about relationship, arguments)  5. Relationship with your children (e.g. frequent conflicts, not showing enough respect)  6. Relationship with parents (e.g. regular conflicts, little or no acceptance)  7. Relationship with other family members (e.g. regular conflicts, little or no acceptance)  8. Free time (e.g. not enough, too much free time)  9. Finances (e.g. large debts, inadequate income)  10. Your health (e.g. regularly ill, chronically ill)  11. School/study (e.g. too difficult, not possible to combine with other tasks)  12. Faith, church or religion (e.g. doubt, conflict with clergyman/parson) | - 0 points (1) - 1-2 points (2) - 2-3 points (3) - 3-4 points (4) - ≥ 5 points (5) | LDI^7,8^ |
| **Body Mass Index (BMI) (kilogram per square meter (kg/m2))** | Weight was measured in kilograms and height was measured in meters.  BMI was calculated as kg/m2. | - <25 kg/m2 (1) - > 25-30 kg/m2 (2) - > 30 kg/m2 (3) | None. |
| **Waist circumference** | Waist circumference was measured standing in centimeters (cm). | - ≤ 80 cm (1) - > 80-90 cm (2) - > 90-100 cm (3) - > 100-110 cm (4) - > 110 cm (5) | None. |
| **Physical activity** | **Commuting** The following data was collected for each item: No. days per week, average time per day and self-reported intensity (slow, moderate, fast).   1. Walking to/from work 2. Biking to/from work   **Physical activity at work or school** The following data was collected for each item: Average time per day.   1. Light and moderate physical activity (sitting or standing, with occasionally walking, for example office work) 2. Vigorous physical activity (walking and frequently lifting heavy weights)   **Household activities**  The following data was collected for each item: No. days per week, average time per day.   1. Light and moderate household activities (standing, for example cooking, washing dished, iron, taking care of children, walking, vacuum cleaning and doing groceries) 2. Vigorous household activities (scrub the floor, walking with heavy groceries)   **Leisure time activities** The following data was collected for each item: No. days per week, average time per day and self-reported intensity (slow, moderate, fast).   1. Walking 2. Biking 3. Gardening 4. Odd jobs   **Sports**  The following data was collected for each item: Sport name, no. days per week, average time per day and self-reported intensity (slow, moderate, fast).   1. Maximum of 4 sports | Every activity was categorized as light, moderate or vigorous based on Metabolic Equivalent Tasks (METs) combined with self-reported intensity. Activities were coded based on Physical Activity Compendium of Ainsworth (2011).^9^ For adults aged 18–55 years, the following cut off values were used: <4.0 Metabolic Equivalent of Tasks (MET) (light intensity), 4.0 to 6.5 MET (moderate intensity), and ≥6.5 MET (vigorous intensity), and for adults aged >55 years, these cut off values were <3.0 MET (light), 3.0 to 5.0 MET (moderate), and ≥5.0 MET (vigorous). The three MET categories were combined with self-reported intensity for each activity, resulting in a combined intensity score ranging from 1 to 9, with 1 being light MET and light self-reported intensity and 9 being vigorous MET and vigorous self-reported intensity. The classification of physical activities according to the combined intensity score was <3 (light intensity), 3 to 6 (moderate intensity), and ≥6 (vigorous intensity). The results from the SQUASH were converted to minutes per week spent for each intensity activity category. Outcomes were presented as:   - Moderate and vigorous physical activity (MVPA) - Vigorous physical activity (VPA) | Short Questionnaire to Assess Health-enhancing physical activity (SQUASH)^10^ and Physical Activity Compendium of Ainsworth (2011)^9^ |

Abbreviations: NOSQ, Nordic Occupational Skin Questionnaire; TRGS, Technische Regeln für Gefahrstoffe; LTE, List of Threatening Experiences; LDI, Long-term Difficulties Inventory; BMI, Body Mass Index; kg/m2, kilogram per square meter; cm, centimeter; METS, Metabolic Equivalent of Tasks; SQUASH, Short Questionnaire to Assess Health-enhancing physical activity; MVPA, moderate and vigorous physical activity; VPA, vigorous physical activity.

**References**

1. Loman, L. & Schuttelaar, M. L. A. Hand eczema and lifestyle factors in the D utch general population: Evidence for smoking, chronic stress, and obesity . *Contact Dermatitis* **86**, 80–88 (2022).

2. Susitaival, P. *et al.* Nordic Occupational Skin Questionnaire (NOSQ-2002): a new tool for surveying occupational skin diseases and exposure. *Contact Dermatitis* **49**, 70–76 (2003).

3. Barbarot, S. *et al.* Epidemiology of atopic dermatitis in adults: Results from an international survey. *Allergy* **73**, 1284–1293 (2018).

4. Behroozy, A. & Keegel, T. G. Wet-work Exposure: A Main Risk Factor for Occupational Hand Dermatitis. *Saf. Health Work* **5**, 175–180 (2014).

5. Bundesanstalt für Arbeitsschutz und Arbeitsmedizin. TRGS 401: Risks resulting from skin contact - identification, assessment, measures, 2008. *Available https//www.baua.de/DE/Angebote/Rechtstexte-und-Technische-Regeln/Regelwerk/TRGS/TRGS-401.html (last accessed 30 April 2021.*

6. Brugha, T., Bebbington, P., Tennant, C. & Hurry, J. The List of Threatening Experiences: a subset of 12 life event categories with considerable long-term contextual threat. *Psychol. Med.* **15**, 189–194 (1985).

7. Rosmalen, J. G. M., Bos, E. H. & de Jonge, P. Validation of the Long-term Difficulties Inventory (LDI) and the List of Threatening Experiences (LTE) as measures of stress in epidemiological population-based cohort studies. *Psychol. Med.* **42**, 2599–2608 (2012).

8. Hendriks, A., Ormel, J. & van de Willige, G. Long-term difficulties measured by a self-report questionnaire and semi-structured interview : a comparison of methods [in Dutch: Langdurige moeilijkheden gemeten volgens zelfbeoordelingsvragenlijst en semi-gestructureerd interview: een theoretische en em. *Gedrag en Gezondh.* **18**, 273–283 (1990).

9. Ainsworth, B. E. *et al.* Compendium of physical activities: classification of energy costs of human physical activities. *Med. Sci. Sports Exerc.* **25**, 71–80 (1993).

10. Wendel-Vos, G. C. W., Schuit, A. J., Saris, W. H. M. & Kromhout, D. Reproducibility and relative validity of the short questionnaire to assess health-enhancing physical activity. *J. Clin. Epidemiol.* **56**, 1163–1169 (2003).
